# Natamycin in Food and Ophthalmology: Knowledge Gaps and Emerging Insights from Zebrafish Models

**DOI:** 10.3390/ph19010086

**Published:** 2026-01-01

**Authors:** Manjunatha Bangeppagari, Pavana Jagadish, Anusha Srinivasa, Woorak Choi, Pragya Tiwari

**Affiliations:** 1 Zebrafish Drug Screening Center, Department of Cell Biology and Molecular Genetics, Sri Devaraj Urs Academy of Higher Education and Research (A Deemed to Be University), Tamaka, Kolar 563103, Karnataka, India; 2 Department of Mechanical Engineering, Korea National University of Transportation (KNUT), 50 Daehak-ro, Chungju-si 27469, Chungcheongbuk-do, Republic of Korea; wrchoi@ut.ac.kr; 3 Department of Horticulture and Life Sciences, Yeungnam University, Gyeongsan 38541, Gyeongsangbuk-do, Republic of Korea

**Keywords:** natamycin, food preservation, microbiome, zebrafish (*Danio rerio*), drug safety

## Abstract

Natamycin, a polyene macrolide antifungal, has long been used as a food preservative and is the only Food and Drug Administration (FDA)-approved topical treatment for fungal keratitis. While its safety is supported by specific ergosterol interaction and minimal systemic absorption, current research mainly focuses on short-term effects, often overlooking long-term, developmental, and microbiome-related impacts. In food applications, questions remain about cumulative exposure and potential disruptions to gut microbiota. For ophthalmology, advanced delivery methods like nanocarriers and hydrogels enhance drug penetration but may alter pharmacokinetics and pose formulation challenges. Regulatory approvals have historically depended on established safe use and limited toxicological data, emphasizing the need for more systematic evaluations. Zebrafish (*Danio rerio*) represent a promising yet underutilized model for addressing significant gaps in research, particularly in the realms of microbiome studies, ocular health, developmental processes, and multigenerational effects. When paired with omics technologies, zebrafish facilitate comprehensive system-level mapping of drug-induced outcomes. This review consolidates existing evidence and positions zebrafish as a vital translational link between in vitro assays, mammalian models, and clinical practice. Additionally, it proposes a framework to ensure the effective and scientifically supported use of natamycin in both food and medicinal applications.

## 1. Introduction

A polyene macrolide antifungal, natamycin, which was originally isolated from *Streptomyces natalensis*. Over time, from serving as a natural food preservative to becoming an essential component of ophthalmic antifungal treatment, natamycin has undergone significant transitions. In the food industry, natamycin is known for its broad-spectrum activity against yeasts and molds, coupled with a promising safety profile that helps maintain food quality. It is generally used to extend the shelf life of products such as cheese, yogurt, and sausages, and is considered generally recognized as safe (GRAS) in many regions [1,2]. Clinically, natamycin is significant as the only Food and Drug Administration (FDA)-approved topical antifungal specifically for treating fungal keratitis, a serious eye condition with limited treatment options [3,4]. Its dual role in food preservation and eye care highlights its unique importance in both industrial and medical fields.

Despite its advantages, natamycin faces pharmacokinetic challenges, particularly in eye treatments. Its low solubility, large molecular size, and limited penetration into the cornea necessitate frequent dosing and restrict access to deeper eye tissues [5,6]. To address these issues, significant advancements have been made through the development of innovative formulations, including nanocarriers, in situ gels, and cell-penetrating peptide conjugates. These improvements improve corneal absorption, prolong retention time, and reduce dosing frequency while maintaining or enhancing safety [3,7]. These developments show how natamycin has evolved from a simple preservative into a more sophisticated therapeutic tool, with ongoing efforts to optimize its application in both food and medical fields.

Of note, the regulatory approval of natamycin by agencies such as the FDA, European Food Safety Authority (EFSA), and Codex Alimentarius is based not only on toxicological data but also on its historical safe use and thorough benefit-risk assessments. Approvals were granted based on the available evidence, expert consensus, and international evaluations, even in the absence of comprehensive long-term toxicology studies [8,9]. These agencies have established maximum residue limits (MRLs) for food applications and continue to monitor new data, balancing ongoing safety with public health needs [10]. This underscores a broader regulatory practice of adaptive approval, where continuous surveillance serves as a substitute for exhaustive pre-market testing for compounds with extensive histories of safe use.

Reviews over the past decade have consistently described natamycin as a compound with low acute toxicity and a favorable safety record, both in food preservation and in ophthalmic therapy [1,3,11]. Yet these reports also reveal important gaps. Data on long-term and cumulative exposure are scarce, and little is known about possible systemic or generational effects. Evidence is similarly limited for the risk of resistance development or for the impact of new delivery systems when used in sensitive populations such as children, pregnant women, or the immunocompromised [12,13]. These omissions are notable given the increasing reliance on advanced ocular formulations and the likelihood that gastrointestinal and ocular microbiota may be influenced by repeated exposure.

**Figure 1:**
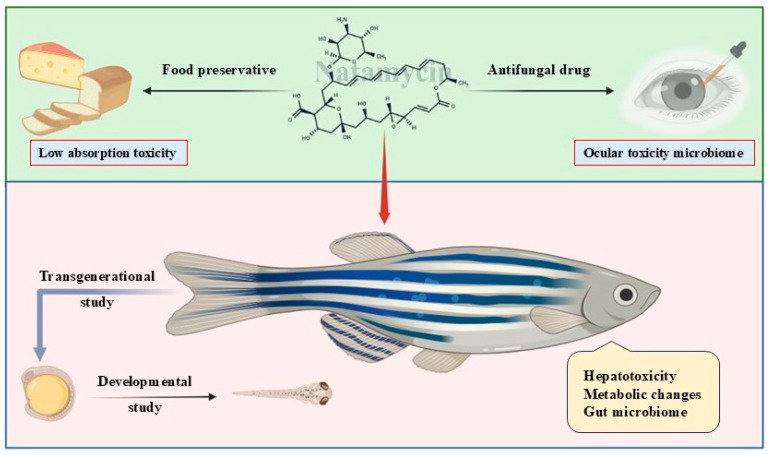
This graphical abstract summarizes how zebrafish models provide emerging toxicological and mechanistic insights into natamycin used in food preservation and ophthalmic therapy. The figure highlights current knowledge gaps—including low-absorption food toxicity, ocular microbiome effects, developmental and transgenerational risks, hepatotoxicity, metabolic changes, and gut microbiome alterations—supporting improved safety evaluation and evidence-based use of natamycin.

The present review brings together available knowledge on natamycin’s mechanism of action, safety profile, and dual applications in food and ophthalmology, while also examining the unresolved areas that restrict a full risk assessment. As illustrated in Figure 1, special attention is given to the zebrafish model, which provides an experimentally tractable system for investigating microbiome alterations, developmental endpoints, and cross-generational effects, as well as the toxicity of novel formulations. By combining evidence from toxicology, microbiology, and pharmacology, zebrafish-based studies have the potential to supply the mechanistic and long-term data required to guide natamycin’s continued safe use in both clinical and industrial settings.

Although food and ophthalmic applications appear distinct, both involve direct human exposure to natamycin under conditions that may influence microbiome stability and long-term safety. A comparative assessment is essential because regulatory evaluations in each sector have historically been independent, despite shared safety determinants such as cumulative exposure, formulation stability, and microbial resistance. Therefore, this review aims to integrate data across food science and ophthalmology to highlight overlapping toxicological considerations and emerging translational insights.

The existing data on natamycin’s safety is not robust enough to establish a definitive Acceptable Daily Intake (ADI), as highlighted by the EFSA. The limitations include inadequate animal study designs and a lack of carcinogenicity studies. Despite these limitations, current exposure levels are considered safe, as dietary intake is significantly lower than the provisional ADI [14]. There are inconsistencies in the enforcement of regulations concerning pharmaceutical excipients, which include natamycin. This is particularly evident in countries like India, where regulatory frameworks are not fully aligned with international standards, leading to potential safety risks [15]. Harmonization with global standards such as those from the FDA and European Medicines Agency (EMA) is necessary to ensure consistent safety and quality across different regions [15]. Natamycin’s poor bioavailability and limited ocular penetration pose significant challenges in its therapeutic application, particularly for ocular infections like fungal keratitis [3,16]. Innovative drug delivery systems, such as niosomal formulations, are being developed to enhance bioavailability and efficacy, thereby addressing some of these challenges [16].

## 2. Mechanism of Action

Natamycin exerts its antifungal activity through a unique mechanism that distinguishes it from other polyene antibiotics. Rather than permeabilizing the fungal cell membrane, natamycin specifically binds to ergosterol, targeting the double bonds located in the sterol’s B-ring [17]. This binding does not result in pore formation, as confirmed by subsequent studies showing that natamycin does not disrupt membrane integrity [18]. Instead, natamycin inhibits ergosterol-dependent membrane transport processes, leading to suppression of amino acid and glucose uptake across the plasma membrane [18]. This functional inhibition arises from natamycin’s ability to sequester ergosterol, impairing the activity of essential membrane proteins involved in nutrient transport. Further evidence from multiple fungal species supports this mechanism: natamycin has been shown to interfere with membrane protein transport, vacuole fusion, and other cellular processes through its highly specific interaction with ergosterol [19].

## 3. Chemical Properties and Pharmacodynamic Profile

Natamycin shows a marked preference for binding fungal ergosterol over mammalian cholesterol, a property that underlies both its antifungal potency and its low mammalian toxicity. Structural studies demonstrate that natamycin aggregates with ergosterol to form highly specific complexes, which effectively sequester the sterol and disrupt its ability to order lipid acyl chains [19,20]. Cholesterol-containing membranes are only minimally affected, except under artificially high concentrations [21,22]. Nuclear magnetic resonance and computational modeling provide further insight, showing a unique “head-to-tail” orientation in natamycin–ergosterol binding, distinct from other polyenes such as amphotericin B [23].

Amphotericin B Binds to ergosterol, forming pores that disrupt membrane integrity, leading to ion leakage and increased reactive oxygen species (ROS) production [24]. This action is cooperative, causing significant membrane perturbation [23].

Natamycin: Interacts with ergosterol in a non-cooperative manner, affecting specific sterol resonances without causing extensive membrane disruption [23]. This selective engagement results in lower toxicity and a different resistance mechanism. Vacuolar Sequestration: Both drugs show enhanced accumulation in vacuoles of resistant strains, suggesting that vacuolar sequestration is a common resistance mechanism [25]. However, AMB (Amphotericin B) resistance is often linked to ergosterol depletion, while natamycin resistance may involve altered sterol composition without significant ergosterol loss [26].

Oxidative Stress Response: AMB induces ROS, which can trigger cell signaling pathways leading to oxidative damage. Resistant strains exhibit mechanisms to mitigate this oxidative stress, such as enhanced antioxidant enzyme activity [24,26].

Unlike amphotericin B, which inserts into membranes and creates pore-like complexes that disrupt permeability, natamycin acts without pore formation [19,23]. Its antifungal activity comes instead from sterol sequestration, disruption of lipid organization, and interference with sterol-dependent protein functions, all while maintaining overall membrane integrity. This non-lytic mechanism explains the compound’s antifungal potency combined with its minimal cytotoxicity in mammalian cells [27].

Despite this favorable pharmacology, natamycin’s therapeutic utility is constrained by its physicochemical properties. The molecule is poorly soluble in water, chemically unstable, and of relatively high molecular weight, all of which limit bioavailability and make it prone to degradation [4,5,6,28]. In ophthalmology, these limitations manifest as weak corneal penetration and the need for frequent dosing to sustain therapeutic concentrations. Researchers have responded by developing new delivery platforms such as cyclodextrin complexes, lipid nanoparticles, cubosomes, bilosomes, nanomicelles, and in situ gelling systems [4,5,6,11,29,30]. These approaches improve stability, enhance solubility, and enable more sustained release of the drug.

Preclinical and clinical work confirms that advanced formulations lead to higher intraocular levels of natamycin, longer residence times, and reduced dosing frequency compared with conventional suspensions. Importantly, they achieve this while maintaining tolerability and minimizing ocular irritation [4,5,11]. Comparable innovations for food applications have not yet been widely studied, but the same principles that enhanced solubility and molecular stabilization are likely to improve natamycin’s persistence and antifungal performance in food matrices.

A clearer distinction among the delivery platforms highlights that each technology carries unique and formulation-specific risks. Lipid nanoparticles (LNPs) pose concerns primarily related to their rheological properties, which may be unsuitable for some applications and may require integration with hydrogels to achieve controlled drug release [31]. Furthermore, emerging Smart Lipids, although offering improved encapsulation and targeted delivery, introduce additional uncertainty due to their more complex structural architecture, which may lead to yet-unknown biocompatibility challenges [32]. In contrast, cyclodextrin complexes are generally recognized for enhancing drug solubility and stability [33,34], but their main safety risk is ototoxicity, particularly when permeation is not adequately controlled, emphasizing the need for careful evaluation of their tissue penetration profiles [33]. Meanwhile, in situ gels differ from both systems in that their potential hazards are tied to environment-driven sol–gel transitions; variations in physiological conditions can influence gelation behavior, potentially affecting the uniformity, consistency, and predictability of drug release [35]. This differentiation illustrates that each platform’s risk profile arises from distinct physicochemical or structural features, underscoring the need for technology-specific safety considerations.

Natamycin forms complexes with ergosterol, compromising membrane integrity, which is crucial for fungal survival [25].

Vacuolar Sequestration: In resistant strains, natamycin accumulates in vacuoles, suggesting a role in stress response and resistance mechanisms [25].

High Osmolarity Glycerol (HOG) Pathway: This pathway regulates responses to environmental stresses, including osmotic and oxidative stress, and is linked to ergosterol biosynthesis [36].

Alkaline pH Response: The sterol homeostasis pathway is essential for growth in alkaline conditions, indicating that natamycin may influence pH adaptation mechanisms [37]. Fungal pathogens exhibit heightened sensitivity to membrane-targeting antifungals like natamycin under alkaline conditions, suggesting that environmental factors can modulate antifungal efficacy [37].

Conversely, while natamycin’s role in disrupting fungal signaling is significant, some studies emphasize the potential for other antifungal agents to induce oxidative or nitrosative stress, which may also contribute to their efficacy against resistant strains [38].

## 4. Toxicity Assessment in Food Applications

Natamycin (E235) has been used as a food preservative for several decades, with regulatory approval based on its favorable acute safety profile and minimal systemic absorption. In the European Union, its application is restricted to the surface treatment of hard, semi-hard, and semi-soft cheeses, with a maximum residue limit (MRL) of 1 mg/dm^2^ applicable to the outer surface. Use beyond a depth of 5 mm is explicitly forbidden [39]. These regulations reflect a precautionary approach aimed at minimizing consumer exposure. However, there is a notable lack of harmonization in global standards. Current literature indicates that the United States and Asian markets employ differing standards, and explicit MRLs are not consistently reported, leading to regulatory asymmetry that could complicate international trade [40,41]. This variability underscores the need for transparent, region-specific evaluations to support global risk assessments.

From a pharmacokinetic standpoint, natamycin exhibits extremely low oral bioavailability due to its high molecular weight, low aqueous solubility, and instability under gastrointestinal conditions [11,42]. Studies in both humans and animals have shown negligible systemic absorption, which has led researchers to focus primarily on topical or mucosal delivery systems [42]. Even in studies involving rabbits and other animal models, systemic distribution following oral administration is undetectable, suggesting that ingested natamycin predominantly remains within the gastrointestinal lumen [11]. This interpretation is further supported by general pharmacokinetic principles associated with poorly soluble, high-molecular-weight compounds [43]. Comparative analyses of other polyene antifungals, such as amphotericin B and its derivatives, reinforce this perspective, demonstrating consistently minimal gastrointestinal absorption across species and emphasizing the need for parenteral administration for effective therapeutic use [12,44,45,46].

The persistence of natamycin within the gastrointestinal tract raises important considerations. Experimental studies suggest that residues of natamycin could interact with commensal gut microbiota, particularly Candida species, exerting selective pressure and fostering the development of resistance traits [12]. Broader investigations of polyene antifungals in murine models have confirmed that chronic antifungal exposure alters both fungal and bacterial community structures, resulting in a reduction in Candida while promoting the growth of other genera such as Aspergillus, Wallemia, and Epicoccum [47]. These shifts in the gut mycobiome have been associated with altered immune responses and an increased susceptibility to inflammatory and allergic diseases, suggesting potential systemic implications even in the absence of systemic absorption. Although direct measurements of natamycin concentrations in the cecum or colon are currently unavailable, these findings highlight a critical knowledge gap regarding its interactions with the gut microbiome and its ecological safety.

In terms of long-term safety, there is a notable lack of chronic feeding studies examining carcinogenicity, reproductive toxicity, or developmental outcomes for natamycin. As illustrated in Table 1 and most existing research is limited to acute toxicity assessments, in vitro cytotoxicity, or short-term exposure models [48,49,50,51]. Data from poison center reports further support its relatively benign acute toxicity profile, showing no instances of severe or fatal cases [52]. However, without systematic long-term studies, the cumulative risk posed by dietary residues remains inadequately defined. This gap is particularly concerning given the increasing focus on microbiome-mediated toxicity and developmental safety within regulatory science.

Importantly, there is no evidence to suggest allergenicity or hypersensitivity reactions linked to foods containing natamycin. Extensive reviews of food allergens consistently exclude natamycin, reinforcing its classification as a non-allergenic additive [1,53,54]. Thus, existing data indicate that concerns regarding food safety are more focused on ecological impacts on gut microbiota and the absence of comprehensive studies on chronic exposure, rather than classical toxicity or allergenicity [55].

When considering natamycin’s toxicological profile in food applications, it is characterized by low systemic absorption and favorable acute safety. However, its potential effects on the gut microbiome and chronic safety remain insufficiently explored. To address these gaps, it is crucial to conduct standardized feeding studies, microbiome analyses, and foster regulatory harmonization across regions to fully validate its ongoing safe use in the global food supply. Thus far, toxicological investigations of natamycin have predominantly been limited in scope, focusing mainly on acute or short-term endpoints, with few systematic long-term or generational studies available. Comparative evidence drawn from related polyene antifungals reveals both the relative safety of natamycin and the well-documented toxicity concerns associated with amphotericin B. A summary of the existing study designs, routes, doses, and outcomes is provided in Table 1, highlighting the predominance of acute and formulation-oriented study, the scarcity of chronic and microbiome-related evaluations, and the lack of standardized cross-species comparisons.

Natamycin is stable under various conditions, but it can degrade when exposed to UV light and high temperatures [56]. Natamycin exhibits low toxicity, making it suitable for food preservation and antifungal applications [1,57]. While its systemic absorption is low, the potential toxicity of any degradation products remains unassessed, indicating a gap in research.

Natamycin, a polyene macrolide antifungal, is used as a food preservative, particularly in dairy products. Its application may expose gastrointestinal flora to selective pressure, potentially leading to resistance development [58]. In vitro studies indicate that subinhibitory concentrations of polyenes can elicit resistance in fungi, suggesting that similar mechanisms could occur in the gut following natamycin consumption [58]. The use of natamycin may alter the composition of microbial communities, although specific metagenomic studies focusing on natamycin’s effects are scarce. The broader context of antibiotic use shows that antibiotics can significantly shift microbial diversity and increase resistance gene prevalence [58,59]. The potential for horizontal gene transfer among resistant strains raises concerns about the long-term implications of natamycin use in food products [58]. In contrast, some studies suggest that non-antibiotic drugs do not significantly select for antibiotic resistance in complex bacterial communities, indicating that the effects of natamycin may not be as pronounced as those observed with traditional antibiotics [60].

Impact of Triazole Fungicides Studies show that exposure to epoxiconazole (EPX) in mice resulted in significant changes in gut microbiota, including increased abundance of harmful bacteria and altered metabolic profiles [61].

Antimicrobial drug residues in food can disrupt the colonization resistance of gut flora, leading to overgrowth of pathogenic microorganisms and increased drug resistance [62].

Model Systems for Assessment: Various model systems, including human flora-associated mice and chemostats, have been used to determine ADIs of antimicrobial residues, indicating that even low levels can affect gut flora [63]. The use of antifungal agents like natamycin in food may exert selective pressure on gastrointestinal fungi, potentially leading to resistance development [58].

**Table 1:** Toxicological Assessment of Natamycin in Food Applications.

Compound/Study	Species/Model	Route of Administration	Dose/Concentration	Duration	Endpoints/Outcomes Assessed	Citations
Natamycin (general use)	Human, animal, in vitro	Topical, oral (poor absorption)	Not specified	Chronic (food/medicine)	Low systemic toxicity, rare resistance, proven food safety	[48,55,64,65]
Natamycin (food preservative)	Human exposure model	Oral (dietary)	Not specified; enhanced with cyclodextrins	Chronic dietary exposure	Effects on gut flora, resistance development, dietary safety thresholds	[55]

## 5. Toxicity Assessment in Ophthalmic Applications

Topical natamycin has been a cornerstone treatment for fungal keratitis for more than fifty years and remains the only FDA-approved antifungal specifically for ophthalmic use. As illustrated in Table 2 and Table 3, both clinical and experimental findings consistently support its strong safety profile. Randomized controlled trials report significantly fewer adverse events in patients treated with natamycin compared to alternative agents such as amphotericin B or voriconazole. No severe corneal epithelial toxicity, vision-threatening complications, or treatment-related perforations have been observed Mild, temporary irritation is the most common side effect, while blurred vision is usually due to underlying keratitis rather than drug toxicity [3,66].

A major limitation of traditional natamycin suspensions is poor corneal penetration and rapid precorneal elimination, which necessitate frequent dosing and may affect compliance. To overcome this, multiple advanced delivery systems have been developed, including nanomicelles, liposomes, transfersomes, niosomes, bilosomes, solid lipid nanoparticles, and in situ gelling formulations [30,70,73]. These platforms prolong residence time, enhance corneal and scleral penetration, and provide sustained drug release. Safety assessments from in vitro and in vivo studies show minimal irritation, low cytotoxicity, and high ocular tolerance [4,70,74]. These improvements enhance compliance and may improve therapeutic outcomes without compromising safety.

Comparative studies further highlight natamycin’s superior tolerability. In direct comparisons, natamycin achieves faster clinical resolution in Fusarium keratitis and shows fewer adverse events than voriconazole, especially in deep stromal infections [75,76]. While amphotericin B is effective for refractory infections, it is associated with greater ocular toxicity, including deep corneal vascularization [76]. Combination regimens using natamycin with voriconazole or other antifungals have shown synergistic benefits without added toxicity [77,78]. Overall, natamycin remains one of the safest and best-studied topical antifungal agents for ophthalmic use [79].

Despite its long history of safe use, some gaps remain. There is limited evidence on natamycin’s impact on the ocular surface microbiome or epithelial barrier integrity, both of which are increasingly recognized as critical to ocular immune homeostasis [80,81,82]. Additionally, safety data in pediatric and immunocompromised patients are limited. Most clinical trials focus on adults with short- or medium-term treatment. Although new formulations—such as water-soluble and nanoparticle-based natamycin—show promising short-term safety in animal models [13]. their long-term effects remain poorly characterized. Longitudinal studies assessing cumulative toxicity and developmental outcomes are needed for high-risk populations.

The first FDA-approved nanocarrier, used for doxorubicin delivery, has paved the way for others like Taxol and Carboplatin [83,84]. These include polymer-drug conjugates and dendrimers, which are designed for targeted delivery and controlled release [83]. Gold and silica nanoparticles are being investigated for their ability to overcome biological barriers and improve drug biodistribution [84]. Combining hydrogels and liposomes for osteoarthritis treatment has shown promise in preclinical studies [85]. Engineered extracellular vesicles are being developed for targeted delivery in hematological disorders [85]. Despite these advancements, challenges remain, including toxicity concerns and regulatory hurdles that may impede the widespread adoption of nanocarriers in clinical settings [86].

Natamycin cubosome nanoparticles have shown increased corneal permeation compared to pure drug suspensions, suggesting improved intraocular delivery [87]. Nanostructured lipid carriers (NLCs) loaded with natamycin demonstrated a 2.5-fold increase in transcorneal permeability, indicating enhanced penetration into ocular tissues [88]. Cubosome formulations of natamycin exhibited sustained drug release, with 84.29% cumulative release over 8 h, which could lead to prolonged drug presence in ocular tissues [87]. NLCs and in situ gelling systems provided sustained release over 24 h, potentially increasing the duration of drug exposure within the eye [88]. PEGylated nanolipid carriers with a gelling system showed improved ocular pharmacokinetic parameters, including enhanced corneal retention and comparable residence time to commercial formulations at lower doses [28]. These formulations demonstrated similar in vivo concentrations in the innermost eye tissues, suggesting effective delivery to deeper ocular regions [28].

Lipid-Based Delivery Systems (LDS): Despite their biocompatibility, the nanoscale nature of LDS can alter drug properties and lead to unexpected toxicological outcomes. The physicochemical characteristics of these systems can significantly impact there in vivo behavior, necessitating comprehensive toxicity assessments to understand their risks fully [89].

Diethylene Glycol and Ethylene Glycol: These excipients are associated with significant toxicity concerns, including renal failure and metabolic acidosis. Their use in pharmaceuticals is tightly regulated, and understanding their mechanism of toxicity is crucial for mitigating risks [90]. The FDA and other regulatory bodies face challenges in setting standards for nanoparticle-based products due to their novel properties. Ethical issues and public opinion also play a role in shaping regulatory frameworks [91]. These systems aim to improve drug stability and reduce adverse reactions. However, regulatory approval requires demonstrating that these systems do not introduce new safety concerns [92].

Conducting large-scale studies that account for variables such as diet can help identify microbiome differences in populations, such as those with autism spectrum disorders (ASD). These studies can track microbiome changes over time, reducing the impact of individual variability and providing insights into causative relationships. Transferring microbiomes into germ-free mice can help establish causality by observing the effects of specific microbial communities on health outcomes [93]. Investigating microbiome effects in natural populations can provide ecological relevance, using observational and manipulative methods to assess behavior and cognitive impacts [94]. Incorporating microbiome assessments in drug development can reveal interactions that affect drug efficacy and safety, thus improving clinical outcomes [95].

The physicochemical characteristics of natamycin indicate that it is less likely to induce significant microbiome disruption compared to smaller-molecule antifungals. As a larger polyene antibiotic, natamycin demonstrates high selectivity by binding specifically to sterols in fungal membranes, thereby minimizing interactions with human cells and commensal microorganisms [23]. This selectivity is largely attributed to its larger molecular structure, which enables targeted antifungal activity while reducing unintended effects on beneficial microbiota [1,56]. Moreover, natamycin binds irreversibly to fungal cell wall components, suppressing fungal growth without substantially affecting human cell membranes [6]. Its sterol interaction is also less cooperative than that observed in smaller antifungal agents, contributing further to its lower toxicity and diminished microbiome disruption potential [23]. The compound’s stability under various environmental conditions and its safe use across applications such as food preservation and topical therapy additionally support its favorable safety profile, reinforcing the notion that natamycin poses limited risk of microbiome disturbance [1,56].

Evidence from related preclinical studies indicates that chronic or repeated dosing of certain drugs can lead to marked histopathological alterations, supporting the relevance of evaluating similar risks for natamycin. For example, prolonged nitrofurantoin exposure in mice produced significant testicular damage, including degeneration of spermatogonia and necrosis within the germ cell layer at higher doses [96]. Likewise, studies of amphotericin B in dogs demonstrated dose-dependent renal histopathological changes, showing that repeated administration can result in substantial tissue injury, particularly with higher cumulative exposure [97]. Although comparable studies specifically examining natamycin’s chronic histopathological effects are currently lacking, these findings suggest that long-term or repeated dosing of polyene antifungals and other drugs can produce measurable organ-level alterations. This pattern highlights the importance of conducting dedicated preclinical investigations to determine whether natamycin may exhibit similar effects under prolonged exposure.

## 6. General Toxicological Considerations Across Applications

Research across both food-related and ophthalmic applications consistently shows that natamycin has an excellent acute safety profile. However, shared knowledge gaps remain, including limited data on chronic exposure, microbiome interactions, and effects in vulnerable populations. These overlapping concerns highlight the need for deeper investigation, as illustrated in Figure 1, and demonstrate the importance of advanced experimental models such as zebrafish for evaluating long-term and population-specific toxicity.

Natamycin is utilized both as a surface preservative in food products and as a topical antifungal agent in ophthalmology. In the context of food, it remains predominantly unabsorbed in the gastrointestinal tract, suggesting potential interactions with the microbiome. In ophthalmology, traditional suspensions face challenges due to low penetration, whereas advanced formulations such as nanocarriers and in situ gels enhance delivery but may also introduce formulation-specific risks. Despite these differing applications, both pathways share several key unresolved issues, including concerns regarding chronic and generational safety, microbiome interactions, formulation-related uncertainties, and the lack of data in vulnerable populations. This underscores the urgent need for more in-depth mechanistic and translational research.

## 7. Knowledge Gaps and Emerging Concerns

Despite the long history of clinical and industrial use, the toxicological evaluation of natamycin remains incomplete. Most available studies focus only on acute or short-term toxicity, leaving major gaps in long-term, chronic, and population-specific safety data. Because natamycin is minimally absorbed when used topically, extensive systemic or generational studies have not historically been prioritized, contributing to these gaps [11,12].

### 7.1. Lack of Long-Term, Chronic, and Generational Studies

Currently, no studies have systematically assessed the carcinogenic, reproductive, or developmental toxicity of natamycin. Although minimal systemic absorption suggests a lower risk, the absence of direct evidence means that chronic or lifetime exposure—particularly through food preservatives—has not been fully evaluated. This gap is important because modern toxicology emphasizes long-term and intergenerational effects.

### 7.2. Unknown Effects on the Microbiome

Antifungal drugs are known to influence both fungal and bacterial microbiota [1]. However, no studies have examined natamycin’s impact on the gut microbiome when consumed in food, or the ocular surface microbiome when used in eye treatments. Although limited absorption is reassuring, potential effects from residual gastrointestinal exposure or advanced ocular formulations remain unstudied.

### 7.3. Safety of Advanced Drug-Delivery Systems

New delivery technologies—such as nanoparticles, liposomes, in situ gels, and ocular inserts—greatly improve solubility, retention, and tissue penetration [98,99,100]. However, these advancements also introduce new uncertainties. For example, nanoparticles may cross epithelial barriers or enter systemic circulation under certain conditions [101]. These modern formulations therefore require dedicated long-term toxicokinetic and safety assessments.

### 7.4. Insufficient Data for Vulnerable Populations

There is little to no toxicological information on natamycin use in children, pregnant or breastfeeding women, immunocompromised patients. These groups may require repeated or prolonged treatment, yet no studies address their specific safety risks. Evidence from unrelated antimicrobial classes suggests that early-life or prenatal exposure can influence immune development or, rarely, contribute to developmental abnormalities [102,103,104], but such findings cannot be applied to natamycin without direct research.

A major issue with commonly used drugs like natamycin is their low bioavailability and poor dispersibility [105,106,107,108]. While the extensively researched composite nanomaterials have solved the above problem [109,110,111], most of their nanocarriers only have delivery effects, and the vector itself often has no biological function.

**Figure 2:**
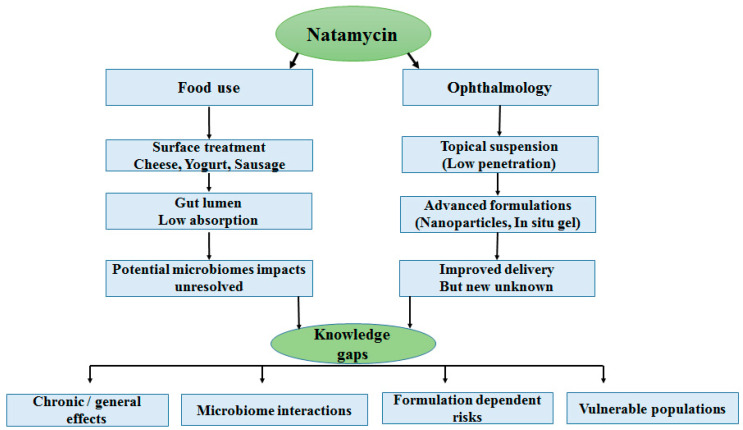
Natamycin across food and ophthalmology: pathways and unresolved questions.

Despite extensive use and general safety recognition, key knowledge gaps persist. As illustrated in Figure 2, data on chronic exposure, developmental outcomes, and microbiome interactions remain sparse, and regulatory standards differ widely among regions. Furthermore, the translational bridge between preclinical safety studies and human outcomes particularly in the context of advanced formulations has yet to be firmly established. Addressing these deficiencies is essential to guide evidence-based use of natamycin in both food and medical sectors.

External factors, including medications, can disrupt ocular development in utero, although specific mechanisms remain unclear. The multifactorial nature of ocular development suggests that any external agent, including natamycin, could potentially interfere with normal embryological processes, leading to developmental anomalies [112]. Ophthalmic medications have been shown to cause teratogenic effects in animal studies, indicating a potential risk to fetal ocular development if absorbed systemically [113]. Early-life environmental exposures, including pharmaceuticals, can lead to persistent changes in immune function. Developmental exposure to certain agents can result in permanent functional differences in the immune system, suggesting that natamycin exposure could potentially have long-term immunomodulatory effects [114]. Nutritional and environmental factors during early development are crucial for programming the immune system. Disruptions during this period can lead to reduced immune responsiveness and increased susceptibility to infections, highlighting the importance of a stable environment for optimal immune development [115].

The ocular surface is home to a stable microbiome, predominantly consisting of genera like Corynebacterium and Staphylococcus, which play a crucial role in immune regulation and protection against pathogens [116,117]. Disruption of this microbiome, potentially through topical medications like natamycin, can lead to increased susceptibility to infections and inflammatory conditions [116]. Studies indicate that perturbations in the ocular microbiome can alter its diversity and composition, which may exacerbate conditions such as dry eye disease and conjunctivitis [117]. The gut microbiome influences ocular health through the “microbiota-gut-eye” axis, where gut dysbiosis is linked to various eye diseases [118,119]. Dietary interventions, including the use of probiotics, can modify gut microbiota composition, potentially benefiting ocular health by enhancing immune responses [119]. The relationship between gut and ocular microbiota suggests that dietary natamycin could indirectly affect ocular health by altering gut microbial profiles [118,119].

Nanoparticle and liposomal formulations of natamycin demonstrate the ability to cross ocular barriers, improving bioavailability and facilitating targeted delivery to ocular tissues, which may also increase the likelihood of entry into systemic circulation [120,121]. Their capacity to penetrate corneal and blood-ocular barriers, along with their sustained-release design, enhances drug retention and reduces administration frequency, supporting therapeutic efficacy while introducing new safety considerations [122,123]. However, chronic exposure to these nanocarriers raises concerns regarding potential immunological reactions and genotoxicity, emphasizing the need for comprehensive in vivo and in vitro safety assessments [122,124]. Importantly, nanoparticle characteristics such as size, shape, and surface properties significantly influence biocompatibility and toxicity profiles, contributing to variability in systemic and ocular responses [121]. Pharmacokinetic data indicate that sensitive groups—including children, pregnant women, and immunocompromised individuals—may exhibit altered absorption and distribution, necessitating individualized dosing approaches [123]. Optimization of formulations for these populations must therefore balance therapeutic benefits with potential adverse effects, ensuring safety while maintaining efficacy [121].

## 8. Zebrafish as a Model System

Zebrafish (*Danio rerio*) have emerged as a robust vertebrate model for toxicology and pharmacology, offering genetic similarities to humans alongside unique experimental advantages such as optical transparency, rapid development, and compatibility with high-throughput in vivo assays. Although their application in antifungal research remains limited, there are notable opportunities for enhancing the safety evaluation of natamycin. [125,126].

To date, studies using zebrafish to assess polyene antifungals have predominantly concentrated on amphotericin B and nystatin, with significantly less focus on natamycin. Research has shown that free amphotericin B exhibits severe embryotoxicity in zebrafish, resulting in organ malformations and lethality at therapeutic concentrations. However, the encapsulation of this drug in biodegradable microspheres successfully mitigated these toxic effects while maintaining its antifungal efficacy [127]. Similar advantages have been observed with nystatin, where encapsulation or combinations with other therapies not only enhanced antifungal activity but also reduced toxicity and resistance [128,129,130]. These findings underscore the utility of zebrafish as a sensitive model for distinguishing between the toxicity of free drugs and the improvements offered by alternative formulations. Nevertheless, there remains a significant gap in the in vivo toxicological characterization of natamycin, as no published studies have utilized zebrafish for its assessment.

Zebrafish are particularly well-suited for studying disruptions in the gut microbiome caused by antifungal agents, which is a critical yet underexplored area concerning natamycin. Previous research has demonstrated that zebrafish gut microbial communities respond significantly to various chemical exposures, including fungicides, antibiotics, and environmental contaminants [131,132,133,134]. For example, exposure to the fungicide imazalil has been shown to alter gut bacterial composition and induce metabolic disturbances, highlighting the model’s sensitivity to antifungal-induced dysbiosis [131]. Established methodologies such as 16S rRNA sequencing, metagenomics, metabolomics, and gut barrier function assays can be effectively employed in studies related to natamycin. This makes zebrafish a valuable model for investigating microbiome-related risks, particularly concerning dietary exposure to natamycin, where residues can persist in the gastrointestinal tract.

Zebrafish serve as a valuable model for studying ocular toxicity due to their retinal and visual system similarities with humans. Research has shown that they can effectively capture various endpoints, including retinal degeneration, structural changes, and functional visual impairments following exposure to toxicants [135,136,137,138]. Behavioral assays, such as optokinetic and visual motor response tests, provide high-throughput functional assessments of vision. However, there are notable limitations: the ocular anatomy of zebrafish differs at the corneal surface, which complicates the evaluation of corneal toxicity and tear film disruption key endpoints in natamycin therapy [135,136,137,138]. Additionally, there is a lack of development in modeling the ocular microbiome, with few studies focusing directly on microbial dysbiosis at the ocular surface in zebrafish. Consequently, while zebrafish can replicate numerous retinal and visual endpoints, methodological innovations will be necessary to tailor them for addressing the unique ocular toxicity issues associated with natamycin.

**Figure 3:**
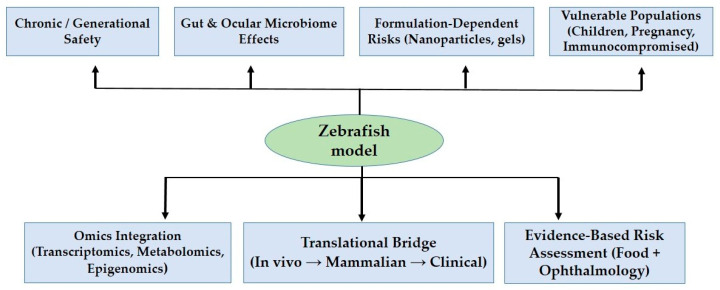
Zebrafish as a bridge for addressing natamycin knowledge gaps.

Zebrafish are extensively utilized to assess the developmental and multigenerational toxicity of antimicrobials, particularly antibiotics. Research has indicated that exposure to antibiotics can lead to maternal and paternal transfer to offspring, with reproductive and developmental impacts that persist across the F1 and F2 generations [139]. Documented endpoints include impaired hatching, morphological malformations, endocrine disruption, and alterations in neurodevelopment [140,141,142,143]. Additionally, antifungal agents such as penthiopyrad and penconazole have been shown to induce mitochondrial dysfunction, bioaccumulation, and enantioselective toxicity in zebrafish embryos [143,144]. These findings highlight zebrafish’s ability to reveal subtle developmental and intergenerational effects, an essential area of inquiry for natamycin, which currently lacks any systematic assessment of chronic or reproductive toxicity. As illustrated in Figure 3, zebrafish present a unique opportunity to address critical gaps in natamycin research, including long-term and generational safety, microbiome-related effects, formulation-dependent risks, and the absence of data concerning vulnerable populations. Serving as a translational bridge between in vitro assays, mammalian studies, and clinical practice, zebrafish models, especially when combined with omics tools, can provide mechanistic and human-relevant insights essential for a unified risk assessment in both food and ophthalmic contexts.

Zebrafish offer a unique combination of genetic and physiological similarities to humans, along with several experimental advantages, such as optical transparency, rapid development, and manageable microbiota [145]. These attributes make zebrafish particularly well-suited for toxicological research. As demonstrated, they can effectively address significant gaps in natamycin research, including chronic and multigenerational safety assessments, interactions with the microbiome, formulation-dependent risks, and impacts on vulnerable populations. The data obtained from zebrafish studies can be integrated with various omics tools, such as transcriptomics, metabolomics, and epigenomics, to elucidate mechanistic pathways and establish a translational bridge connecting in vitro assays, mammalian studies, and clinical data. This system-level approach fosters the development of a cohesive, evidence-based risk assessment framework for natamycin applicable to both food safety and ophthalmological contexts.

Zebrafish models provide a unique opportunity to bridge food and ophthalmic safety assessments by enabling the simultaneous evaluation of mucosal toxicity and epithelial barrier effects. Their transparent embryos and rapid development allow high-throughput screening of chemical-induced mucosal damage, offering early indicators of potential adverse effects relevant to both ingestion and ocular exposure [146,147]. The ability to visualize mucosal injury through fluorescent transgenic lines further strengthens their utility for detecting subtle toxicity patterns that may be shared across food-contact and ophthalmic applications [147]. In parallel, zebrafish possess epithelial barrier structures comparable to those found in humans, allowing researchers to assess how compounds such as natamycin may penetrate, alter, or disrupt epithelial integrity [137,138]. Molecular assays in zebrafish also enable characterization of gene expression changes associated with epithelial barrier function, providing mechanistic insight into potential disruptions [148]. By integrating these capabilities, zebrafish serve as a versatile platform for evaluating both ocular and systemic toxicity, effectively bridging food safety and ophthalmic research domains and supporting comprehensive safety assessments of natamycin [137,138,139,140,141,142,143,144,145,146,147,148].

Zebrafish exhibit substantial but not complete metabolic similarities to mammals, and these differences must be considered when interpreting natamycin toxicity outcomes. Although many cytochrome P450 orthologs are conserved, zebrafish demonstrate distinct metabolic capabilities compared to mammals [149]. They are able to perform key phase I and phase II reactions—such as oxidation, N-demethylation, and glucuronidation—but the relative abundance of metabolites is often lower than in mammalian systems, with parent compounds frequently remaining predominant [150]. These metabolic distinctions may influence natamycin bioavailability, clearance, and tissue accumulation in zebrafish, potentially altering toxicity profiles compared to mammals.

To address these cross-species differences experimentally, several strategies are available. One approach is the creation of “humanized” zebrafish lines expressing human metabolic enzymes, which improves translational relevance by more closely mimicking human xenobiotic processing [151]. Additionally, comparative metabolite profiling at different developmental stages can clarify age-dependent metabolic variations that may affect natamycin toxicity [152]. Zebrafish can also be effectively positioned as an intermediate model between cell-based assays and mammalian systems, enabling early identification of metabolic liabilities while ensuring alignment with later in vivo studies [153].

Despite their advantages, zebrafish models encounter several translational challenges. Variations in drug metabolism, organ system complexity, and pharmacokinetics compared to mammals may impact toxicological outcomes [135,154,155]. Zebrafish embryotoxicity assays can sometimes overestimate developmental risk, leading to false positives that do not always align with mammalian data [66]. Additionally, methodological variability and a lack of standardized assays impede reproducibility and regulatory acceptance [155]. These limitations are particularly pertinent for natamycin, which has negligible systemic absorption in mammals; as a result, zebrafish models may overpredict systemic toxicity if exposure conditions do not accurately reflect realistic human dosing. Bridging these gaps will necessitate side-by-side validation studies with mammalian models, the standardization of exposure protocols, and the integration of zebrafish endpoints with omics-based biomarkers and human-relevant data.

Zebrafish provide a powerful and versatile model to address the current gaps in natamycin research. Their suitability for long-term and transgenerational studies enables the assessment of chronic and generational effects of natamycin exposure, supported by their rapid development and high fecundity, which allow multi-generational observations within a short timeframe [125]. Zebrafish also offer a unique platform for investigating microbiome-related effects, enabling controlled evaluation of how natamycin interacts with the gut microbiota. Previous research has demonstrated that xenobiotics can modulate microbial composition and potentially induce dysbiosis, emphasizing the importance of such investigations in toxicity assessment [156]. Furthermore, their transparent embryos allow real-time monitoring of formulation-dependent risks, including the toxicological differences between conventional natamycin preparations and advanced delivery systems. Studies have shown that formulation significantly affects bioavailability and toxicity, highlighting the necessity of zebrafish-based comparative testing [118,157]. Zebrafish can additionally simulate exposures in vulnerable populations, including developing organisms or immunocompromised conditions, offering insights into population-specific risks that remain understudied in natamycin safety assessment [125].

## 9. Future Directions

Despite its long history of safe use both clinically and industrially, substantial gaps still exist in the toxicological characterization of natamycin. Future research should utilize innovative model systems, advanced analytical tools, and integrative frameworks to generate mechanistic, long-term, and translationally relevant safety data. The use of zebrafish models, paired with state-of-the-art omics technologies, presents a particularly promising approach to meet these needs.

The integration of zebrafish assays with transcriptomics, metabolomics, and epigenomics represents a robust strategy for elucidating toxicity pathways at a systems level. Previous research involving antibiotics, antifungals, and environmental toxicants has demonstrated that multi-omics approaches can identify differentially expressed genes, altered metabolites, and heritable regulatory changes in exposed zebrafish [140,158,159,160,161,162,163]. Transcriptomic profiling (RNA-seq) can reveal disruptions in metabolic pathways, endocrine signaling, or developmental processes, while metabolomics uncovers perturbations in lipid and energy metabolism. Additionally, epigenomics captures transgenerational molecular imprints. By integrating these datasets, researchers can identify hub genes, metabolic bottlenecks, and regulatory nodes that contribute to adverse outcomes [140,158]. Employing these omics-enabled zebrafish platforms to study natamycin will be crucial for mapping mechanistic toxicity pathways and enhancing alignment with human health risk assessments.

To address critical gaps in chronic, developmental, and microbiome-related endpoints, zebrafish studies should implement longitudinal, multigenerational designs. Developmental toxicity can be evaluated by exposing embryos and larvae during key developmental windows, followed by assessments of morphological, behavioral, and molecular endpoints into adulthood and across subsequent generations. Concurrent microbiome studies employing 16S rRNA sequencing, metagenomics, and metabolomics can effectively capture shifts in gut microbial composition, diversity, and host-microbe interactions induced by natamycin [164]. Notably, repeated or lifelong exposure paradigms should simulate both food-related (low-dose, chronic) and ophthalmic (topical, intermittent, higher concentration) scenarios. Integrating these designs with omics analyses will facilitate the identification of persistent, transgenerational, and microbiome-mediated effects that may be overlooked in traditional acute studies.

Comparative analyses are essential for differentiating the class effects of polyene antifungals from the specific actions of natamycin. Both in vitro and clinical data indicate that natamycin demonstrates unique efficacy against Fusarium keratitis when compared to azoles and amphotericin B [75,165,166]. This framework of comparison should also be applied to toxicity endpoints. For instance, studies on cytotoxicity and ocular irritation could help determine whether the observed effects are related to the polyene class as a whole or are specific to natamycin formulations [12,167]. Additionally, combination studies (such as natamycin paired with chlorhexidine or voriconazole) provide valuable insights into synergistic versus compound-specific interactions [78,166]. Conducting systematic comparative toxicology across various antifungals, particularly through zebrafish and mammalian models, will enhance the translational significance of safety assessments for natamycin.

A unified risk assessment framework is essential for integrating data from food safety and ophthalmology. This framework should encompass chemical, microbiological, toxicological, and clinical endpoints through a tiered, systems-based approach [168,169]. Initiatives focused on next-generation risk assessment, such as RISK-HUNT3R, highlight the importance of human-relevant data, computational toxicology, and systems biology to connect preclinical, clinical, and real-world exposures [170]. By applying these methodologies to natamycin, we can achieve harmonization of data across dietary and therapeutic contexts. Furthermore, conducting risk–benefit analyses using health metrics like Disability-Adjusted Life Years (DALYs) would facilitate transparent decision-making [169]. Importantly, engaging stakeholders, fostering cross-sector collaboration, and employing iterative validation through case studies are crucial for ensuring scientific rigor and regulatory acceptance [171,172].

Integrating multi-omics platforms in zebrafish offers a practical systems-toxicology approach to define mechanistic toxicity pathways relevant to natamycin. Transcriptomics provides early, high-throughput detection of molecular perturbations, while proteomics and metabolomics supply functional and biochemical confirmation, including gut–liver axis and mitochondrial alterations [173,174,175]. Time-resolved, dose-dependent sampling anchored to apical phenotypes enables the identification of molecular initiating events and downstream key events within an AOP framework [174,176]. Although natamycin-specific mechanistic evidence in zebrafish is lacking, applying early transcriptomic screening followed by targeted proteomic and metabolomic validation can reveal pathway-level disruptions [173,175]. The integration of these datasets through network modeling facilitates translation by identifying conserved pathways and supporting cross-species validation in mammalian or human-relevant systems to strengthen human health risk assessment [174].

Longitudinal and multigenerational zebrafish studies can be designed by exposing embryos, juveniles, and adults across the F0–F2 generations. Chronic dietary exposure can be modeled using continuous low-dose waterborne or feed-based natamycin that reflects food residue levels, while ophthalmic exposure can be simulated using short, repeated higher-concentration immersion pulses that mimic topical clinical dosing. Key endpoints should include growth, reproduction, development, microbiome composition, and molecular changes. Tracking these outcomes across generations will help determine whether natamycin induces cumulative or heritable effects under both dietary and ophthalmic exposure scenarios.

## 10. Conclusions

Natamycin remains a uniquely important compound as both a food preservative and the only FDA-approved topical ophthalmic antifungal. Despite its long history of safe use, critical gaps persist—including the absence of chronic, multigenerational, and microbiome-focused evaluations, uncertainties introduced by advanced formulations, and limited data for vulnerable populations. Addressing these gaps requires modern experimental approaches and more predictive model systems. Zebrafish offer a valuable complementary platform with advantages in transparency, genetic accessibility, and high-throughput toxicity detection, enabling early identification of neurotoxic, hepatotoxic, and cardiotoxic effects [135,154,177]. While embryonic assays may occasionally overestimate developmental risks, they remain powerful tools for mechanistic investigations and hazard screening when standardized and transparently reported [66]. Moving forward, research should prioritize improved delivery systems to enhance natamycin’s solubility and bioavailability [178,179], deeper characterization of antifungal mechanisms and resistance pathways [180,181], and sustainable production strategies for higher yield and purity [173]. Strengthened toxicological and microbiome-focused evaluations, together with real-world monitoring, will be essential to maintain confidence in natamycin’s applications. In summary, although natamycin’s safety profile is well established, updated and mechanistically informed toxicological assessments integrating zebrafish models, omics-based methods, and modern risk-assessment frameworks are essential to ensure its continued safe use in both food and ophthalmic medicine.

## Figures and Tables

**Table 2:** Toxicological Profile of Natamycin in Ophthalmic/Medical Use.

Compound/Study	Species/Model	Route of Administration	Dose/Concentration	Duration	Endpoints/Outcomes Assessed	Citations
Natamycin (semisynthetic amides)	Mice, human cell lines	intravenous injection, invitro (cell culture medium)	Not specified (in vivo); MICs in vitro	Acute (in vivo), short-term (in vitro)	LD50/ED50 ratio, cytotoxicity, antiproliferative activity, antifungal efficacy	[12]
Natamycin (nanoparticle delivery)	Mice, in vitro	Ocular (topical)	Not specified	Short-term	Cytotoxicity, ocular tolerance, systemic safety, anti-inflammatory effects	[13]
Amphotericin B, Nystatin	Human, animal, in vitro	Parenteral (AmB), oral/topical (Nys)	3–4 mg/kg/day (AmB liposomal)	Variable	Nephrotoxicity, hemolytic toxicity, antifungal efficacy, resistance	[48,64,65]

**Table 3:** Clinical and Preclinical Evidence for Natamycin in Ocular Therapeutics.

Category	Study/Key Details	Design/Context	Outcomes/Findings	References
Clinical Trial	Mycotic Ulcer Treatment Trial I	Randomized, double-masked, multicenter (*n* = 120)	No significant difference vs. voriconazole in visual acuity at 3 months (*p* = 0.29); similar perforation rates (9/60 vs. 10/60)	[67]
Clinical Trial	Systematic Review & Meta-analysis	7 RCTs, 804 participants; comparison with econazole, chlorhexidine, voriconazole, fluconazole	Better outcomes vs. voriconazole (−0.18 logMAR; *p* = 0.006); especially superior in *Fusarium* cases (−0.41 logMAR; *p* < 0.001)	[68]
Clinical Trial	Cochrane Meta-analysis	8 RCTs, 793 participants; overall treatment effectiveness	Improved visual acuity vs. voriconazole (WMD 0.13); lower keratoplasty risk (RR 1.89 for voriconazole)	[69]
Preclinical Study	Niosomal Delivery System	In vitro/in vivo rabbit model; natamycin-loaded niosomes with ketorolac gel	96.43% entrapment; prolonged release (40.96–77.49% over 24 h); enhanced corneal penetration	[70]
Preclinical Study	Susceptibility Analysis	MIC testing on trial isolates (*n* = 221)	Higher natamycin MIC correlated with increased scar size (+0.29 mm) and higher perforation risk (OR 2.41)	[71]
Preclinical/Review	Drug Development Review	Review of formulation limits and bioavailability	<5% bioavailability; molecular mass > 500 Da restricts penetration; need for advanced delivery systems	[3]
Overall Significance	FDA Approval Status	Clinical importance	Only FDA-approved topical antifungal for fungal keratitis	[3]
Overall Significance	Preferred Drug for Filamentous Fungi	Clinical importance	Most effective for filamentous fungi, especially *Fusarium*	[72]
Overall Significance	Safety Profile	Clinical importance	Excellent safety with fewer adverse effects	[3]

## Data Availability

No new experimental data were created.

## Abbreviations

FDA	Food and Drug Administration
GRAS	Generally recognized as safe
MRLs	Maximum residue limits
EFSA	European Food Safety Authority
ADI	Acceptable daily intake
EMA	European Medicines Agency
ROS	Reactive oxygen species
AMB	Amphotericin B
NLCs	Nanostructured lipid carriers
LDS	Lipid-based delivery systems
